# The Danger Signal Adenosine Induces Persistence of Chlamydial Infection through Stimulation of A2b Receptors

**DOI:** 10.1371/journal.pone.0008299

**Published:** 2009-12-14

**Authors:** Matthew A. Pettengill, Verissa W. Lam, David M. Ojcius

**Affiliations:** Health Sciences Research Institute and School of Natural Sciences, University of California Merced, Merced, California, United States of America; University Paris Sud, France

## Abstract

Infections with intracellular bacteria such as chlamydiae affect the majority of the world population. Infected tissue inflammation and granuloma formation help contain the short-term expansion of the invading pathogen, leading also to local tissue damage and hypoxia. However, the effects of key aspects of damaged inflamed tissues and hypoxia on continued infection with intracellular bacteria remain unknown. We find that development of *Chlamydia trachomatis* is reversibly retarded by prolonged exposure of infected cells to extracellular adenosine, a hallmark of hypoxia and advanced inflammation. In epithelial cells, this effect was mediated by the A2b adenosine receptor, unique in the adenosine receptor family for having a hypoxia-inducible factor (HIF1-α) binding site at its promoter region, and was dependent on an increase in the intracellular cAMP levels, but was independent of cAMP-dependent protein kinase (PKA). Further study of adenosine receptor signaling during intracellular bacterial infection could lead to breakthroughs in our understanding of persistent infections with these ubiquitous pathogens.

## Introduction

*Chlamydia trachomatis* species are the leading cause of bacterial sexually transmitted infection and preventable blindness [1]. Chlamydiae are obligate intracellular bacteria which mature and reproduce through a unique biphasic developmental cycle, infecting as metabolically inactive elementary bodies (EBs) and maturing into metabolically active but non-infectious reticulate bodies (RBs), which proliferate before condensing back into infectious EBs to complete the cycle [2]–[4]. Much of the pathology caused during *Chlamydia* infection is due to the inflammatory response it invokes from the host. The initial innate immune response recruits inflammatory cells and T cells necessary for resolution of infection, which depends heavily on production of IL-12 and IFN-γ [5]. Besides the dominant role of IFN-γ and a TH1 response for clearance of infection in vivo, IFN-γ has been implicated in the development of persistent *Chlamydia* infection in vitro [6], while other factors that could contribute to persistent pathogenic infection may be released from the infected tissue rather than uninfected immune cells.

Adenosine has been suggested to function as a ‘STOP’ signal for the immune response during excessive inflammatory conditions which threaten not only the pathogen, but also the infected or neighboring tissues [7]. Extracellular adenosine signals through four characterized adenosine receptors (ARs) – A1, A2a, A2b and A3 – of which only the A2b AR is upregulated during hypoxia [8]. In fact, A2b contains a hypoxia inducible factor (HIF-1a) binding site in its promoter region [9], suggesting that A2b may play a key role in the response to extracellular adenosine under hypoxic conditions, whereas the A2a AR may dominate the response during inflammation in the absence of hypoxia. While increasing attention has been paid to the effects of extracellular adenosine on the inflammatory response [7], [10], no studies have been performed to evaluate the possible effects of adenosine directly on the infected cell or on latency of infection. We therefore investigated a key aspect of inflammation and hypoxia, prolonged exposure to extracellular adenosine on infection by an intracellular pathogen, *C. trachomatis* (Figure S1).

## Results

To test whether adenosine exposure could affect the outcome of C. trachomatis infection in epithelial cells, we first measured the response to 5′-(N-ethylcarboxamido) adenosine (NECA), which stimulates G_s_-coupled ARs. Cervical epithelial cells (HeLa 229) infected with *C. trachomatis* serovar LGV L2 were treated with NECA, which caused a 90% reduction in reinfectious chlamydiae within a few hours of addition. However, reinfectious bacteria recovered to levels of untreated host-cells by the end of the developmental cycle (Figure S2). This suggests that chlamydial development was substantially interrupted for short times after G_s_-coupled AR stimulation, but that the effect was reversible, since chlamydial viability was not significantly affected.

Cyclic AMP (cAMP) levels were measured in HeLa cells in response to adenosine alone, or adenosine added after an inhibitor of adenosine deaminase, erythro-9-(2-hydroxy-3-nonyl) adenine (EHNA). cAMP levels in HeLa cells more than doubled within 30 minutes of adenosine stimulation, and remained elevated in the presence of EHNA, whereas cAMP levels receded back to the level of unstimulated cells six hours in the absence of adenosine deaminase inhibition (Figure S3). Thus G_s_-coupled, and not G_i_-coupled, adenosine receptor stimulation dominates during exposure to 50 µM adenosine in HeLa cells. The expression of all four adenosine receptors in HeLa cells was confirmed by PCR, which showed, among the G_s_-coupled receptors, markedly higher levels of A2b than A2a (Figure S4).

**Figure 1:**
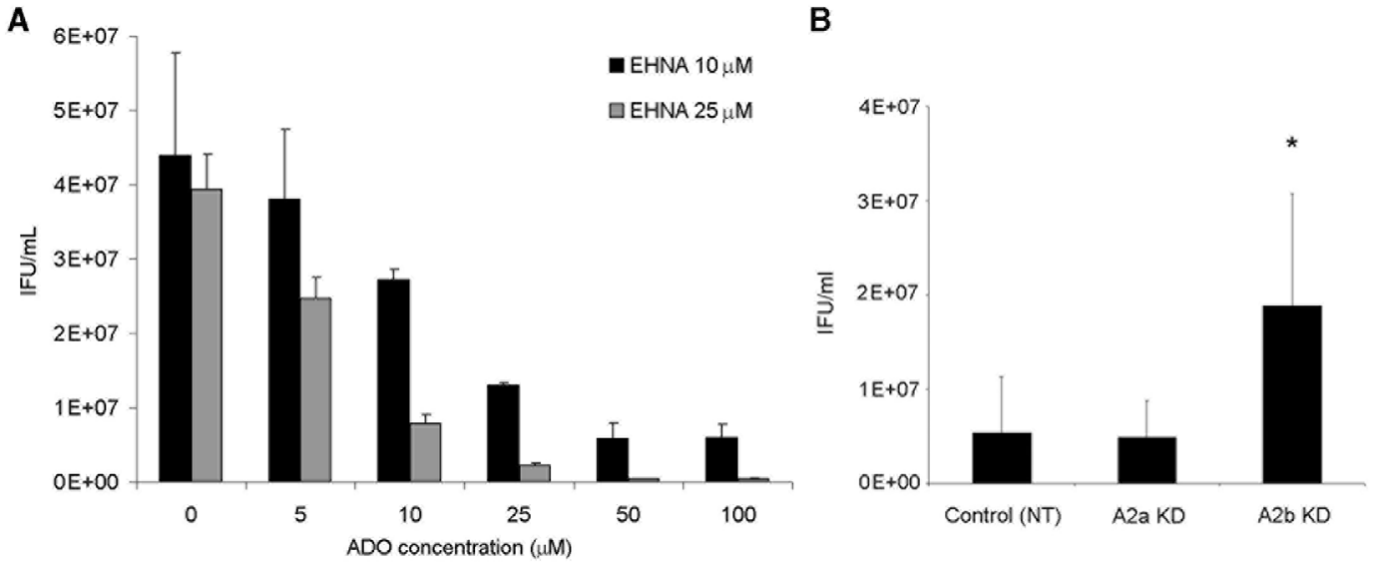
Extended exposure to extracellular adenosine (ADO) causes a dose-dependent reduction in chlamydial development via the A2b adenosine receptor. (**A**) HeLa cells were infected with *C. trachomatis* serovar L2 at an MOI of 1, followed by treatment with the adenosine deaminase inhibitor, EHNA, and ADO at indicated concentrations at 1 and 20 hours post infection (hpi). Samples were harvested at 42 hpi for quantification of reinfectious yield (IFU/ml) on new cell monolayers. The values show averages and S.D. from 3 samples of a representative experiment, and give results obtained from three independent experiments. (**B**) HeLa cell knock-downs (KDs) were produced with non-targeted (Control), A2a-targeted (A2a KD) or A2b-targeted (A2b KD) shRNA and levels of target KD verified to be decreased at least 50% by qPCR. Cells were infected and harvested as (A), with treatment of 25 µM EHNA and 50 µM ADO at 1 and 20 hpi. The values shown are averages and S.D. of values from 4 independent experiments. (n = 4, *, *P*<0.05, for treated cells in A2b KD compared to nontarget control.)

To investigate the outcome of *C. trachomatis* infection in epithelial cells during prolonged exposure to adenosine, we treated epithelial cells with EHNA before addition of adenosine. Synergistic and concentration-dependent effects of EHNA and adenosine co-incubation on infected epithelial cells resulted in ≥90% or greater reduction in the yield of reinfectious chlamydiae at moderate adenosine concentrations (50 µM) (Figure 1A). Similar results were also achieved during infection with the more clinically-prevalent *C. trachomatis* serovar D (Figure S5).

To identify the specific G_s_-coupled ARs involved in adenosine-mediated inhibition of chlamydial development, shRNA-encoding lentiviruses targeting the two human G_s_-coupled ARs, A2a and A2b, were used for depleting the receptors in epithelial cells. Cells depleted of A2b mRNA allowed significantly more robust chlamydial growth in the presence of EHNA and adenosine, compared to control cells transduced with non-targeted shRNA-encoding lentiviruses. In a representative experiment, the reinfectious yield more than doubled in A2b-depleted cells (Figure 1B). In contrast, A2a depletion did not enhance chlamydial growth during extended adenosine exposure. mRNA quantification by qPCR was used to verify greater than 50% mRNA reduction for each target AR in each depletion experiment, performed separately on three occasions.

**Figure 2:**
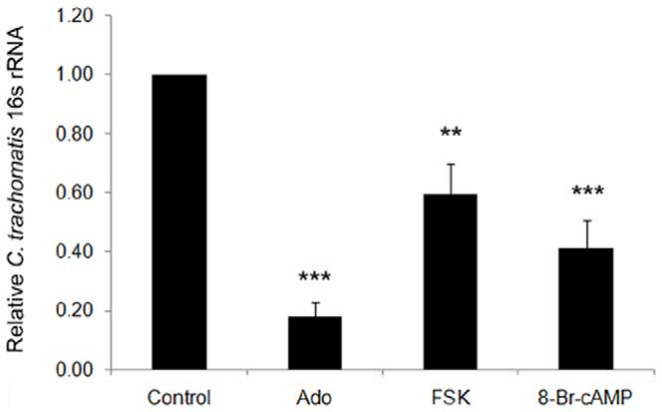
Repeated exposure to cell permeable cAMP analog or cAMP elevating agents causes a reduction in chlamydial development. HeLa cells were infected with *C. trachomatis* serovar L2 at an MOI of 1, followed by treatment with adenosine (100 µM), forskolin (FSK, 10 µM), or 8-Br-cAMP (100 µM) starting at 1 hour post infection (hpi) every half-hour through 6 hpi (10 additions). Total RNA was harvested at 24 hpi for quantification of chlamydial 16s rRNA production using qPCR as indicated in methods. The values shown are relative to control values for each experiment, and are averages and S.D. from 3 independent experiments. (n = 3, **, *P*<0.0005, ***, *P*<0.0001 for treated cells compared to untreated control.)

Additionally, administration of adenosine alone (100 µM) repeatedly led to a greater than 80% reduction in *C. trachomatis* 16s rRNA generation by 24 hours post-infection (hpi) compared to controls, which received vehicle alone over the same time period (Figure 2). Thus prolonged exposure to extracellular adenosine dramatically altered the growth of *Chlamydia* in epithelial cells. Significant reductions were also achieved over this time course using a cell-permeable cAMP analog, 8-bromoadenosine-3′, 5′-cyclic monophosphate (8-Br-cAMP), or forskolin (FSK), an activator of adenylyl cyclase (Figure 2), suggesting that intracellular cAMP increase downstream of adenosine receptor ligation is responsible for the effects on chlamydial development. Pretreatment of host cells with cell-permeant cAMP analogs before infection had previously been reported to reduce uptake of *C. trachomatis* [11], although the receptor stimulated was not determined. However, treatment with 8-Br-cAMP starting at 1 hpi did not reduce the percentage of inclusion-bearing cells (data not shown).

**Figure 3:**
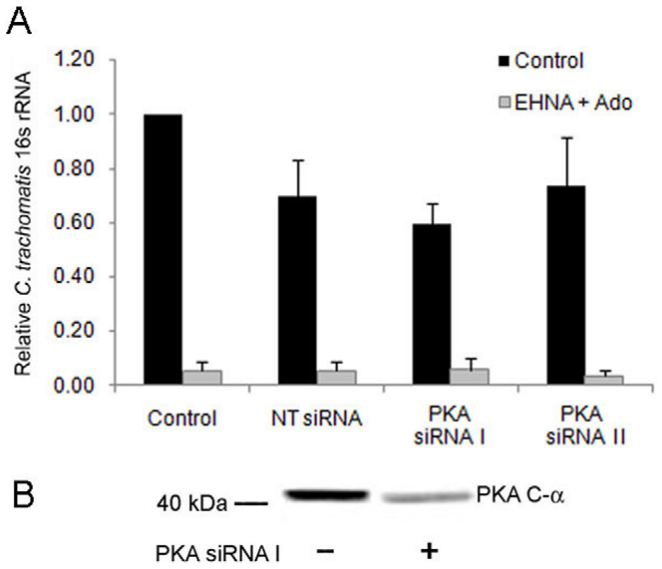
Effects of adenosine on *C. trachomatis* growth are independent of PKA. (A) HeLa cells were transfected PKA C-α targeted siRNA, then infected 30 hours post transfection with *C. trachomatis,* and treated with EHNA (25 µM) and adenosine (50 µM) 1 hpi. Total RNA was harvested at 24 hpi for quantification of chlamydial 16s rRNA production using qPCR as indicated in Materials and Methods. The values shown are relative to control values for each experiment, and are averages and S.D. from 3 independent experiments. (B) Western blot confirmation of PKA C-α depletion by PKA siRNA but not nontarget siRNA. Western blot was performed twice on two separate cell samples.

Host responses resulting from an increase in the intracellular cAMP concentration are often mediated by cAMP-dependent protein kinase (PKA). We therefore depleted HeLa cells of the catalytic subunit of PKA (PKA C-α) using siRNA, and evaluated whether diminished chlamydial growth due to adenosine treatment was dependent on this signaling pathway. In cells with significantly reduced levels of PKA C-α mRNA (Figure S6), prolonged exposure to adenosine diminished chlamydial development as effectively as in cells treated with non-targeted siRNA (Figure 3A), suggesting a PKA-independent mechanism for adenosine-induced growth retardation of *C. trachomatis*. Depletion of PKA C-α protein in HeLa cells was also confirmed by Western blot (Figure 3B); and pharmacological inhibition of PKA with N-[2-(p-bromocinnamylamino)ethyl]-5-isoquinolinesulfonamide (H89) did not alter the functional response to adenosine with respect to infection (data not shown).

We also investigated whether cycloheximide, an inhibitor of eukaryotic but not prokaryotic protein synthesis, could prevent the effect of adenosine of chlamydial development. Cycloheximide at 1 µg/ml, which enhances slightly chlamydial development under normal infection conditions, did not allow significantly more production of chlamydial 16s rRNA during treatment with adenosine (Figure S7), suggesting that production of new host-cell protein does not play a major role in the observed limitation of chlamydial development.

**Figure 4:**
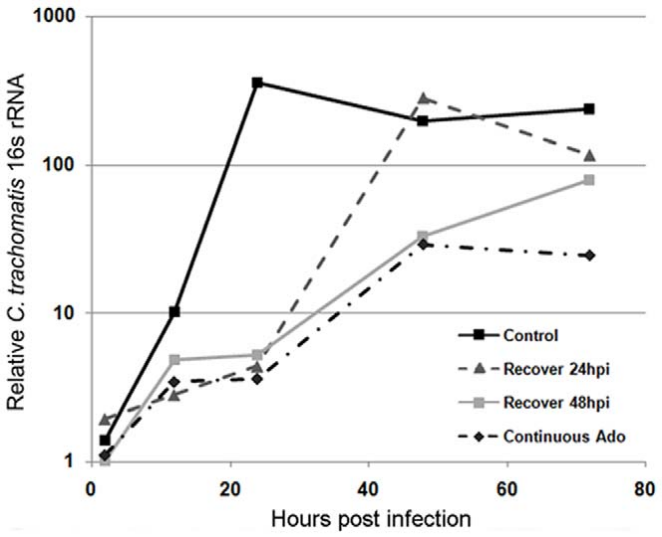
Exposure to adenosine induces a reversible block in chlamydial development. HeLa cells were infected with *C. trachomatis* serovar L2 at an MOI of 1, followed by treatment with an equivalent volume of vehicle alone (control) or 25 µM EHNA and 50 µM adenosine at 1 hpi (non-control samples), 24 hpi (48hpi recovery; and Continuous Ado), and 48 hpi (Continuous Ado only). Media was refreshed in all samples at 24 and 48 hpi, and EHNA and adenosine added again to the samples indicated. Total RNA was harvested at 24 hpi for quantification of chlamydial 16s rRNA production using qPCR as indicated in methods. The values shown are averages plus S.D. of 3 independent experiments.

To show that reduction in growth was not due to death of the chlamydiae, infected cells were treated with EHNA and adenosine for extended periods of time. Lower levels of reinfectious chlamydiae were harvested following these conditions; however, removal of EHNA and adenosine and replenishment with cell-culture medium allowed rapid recovery of chlamydial 16s rRNA production (Figure 4) and reinfectious bacteria (Figure S8). Chlamydial 16s rRNA generation is higher for metabolically active RBs compared to EBs, and moderate levels of 16s rRNA at late time points, along with more significantly diminished reinfectious yield, indicates enduring chlamydial activity with incomplete development for samples with continued exposure to adenosine.

**Figure 5:**
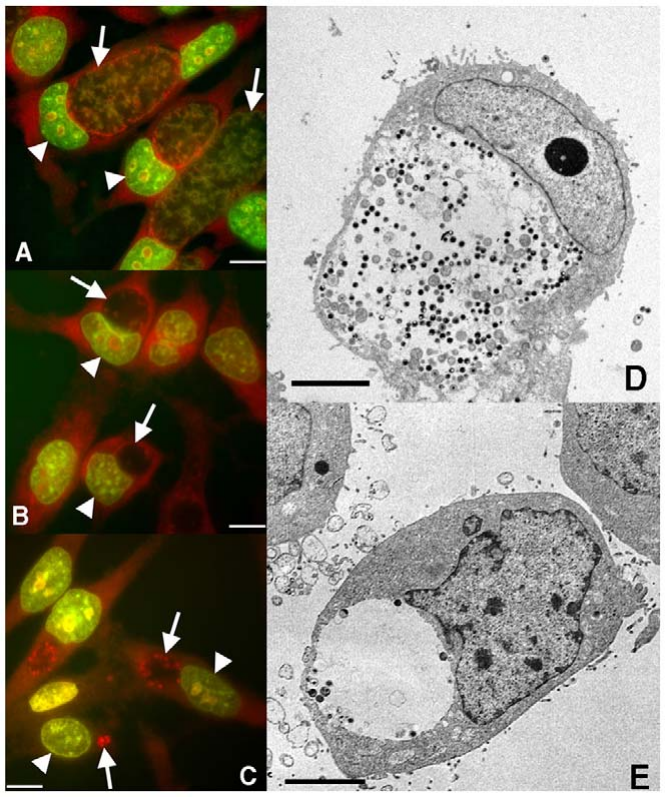
Extended exposure to adenosine causes modification of *Chlamydia* inclusions, tending to moderately sized inclusions with few chlamydiae, compared to smaller-sized inclusions with larger chlamydial forms following treatment with IFN-γ. HeLa cells were infected with *C. trachomatis* serovar L2 at an MOI of 1.0. Samples were (**A, D**) untreated, (**B, E**) treated with 25 µM EHNA and 50 µM ADO at 1 and 20 hpi, or (**C**) treated with 5 ng/ml IFN-γ at 1 hpi. Staining with Acridine Orange was performed as described in Methods with fixation at 42 hpi. Red staining corresponds to RNA localization, while green staining corresponds to DNA. Arrows, *Chlamydia* inclusion; arrowheads, host-cell nuclei. Representative of three experiments, scale bars 10 µm. Electron microscopy samples were also fixed at 42 hpi and prepared for transmission electron microscopy as described in supporting material. Scale bars, 5 µm.

Fluorescence microscopy of EHNA and adenosine-stimulated *C. trachomatis*-infected cells revealed inclusions which were consistently smaller than those in untreated cells, and were largely devoid of chlamydial bodies (Figure 5). Acridine orange staining allows differentiation of chlamydial populations as primarily RBs (RNA∶DNA ratio 3∶1) or primarily EBs (RNA∶DNA ratio 1∶1), with a peak emission in green when bound to DNA and red when bound to RNA [12], and also observation of the localization of RBs and EBs. In the absence of treatment, chlamydiae are rich in DNA (mostly EBs) at 42 hpi, and are found mostly within the inclusions, consistent with other studies [13] (Figure 5A). However, chlamydial populations in infected cells treated with EHNA and adenosine consisted of small numbers of RNA-rich RBs, as well as some EBs, scattered along the periphery of the inclusions (Figure 5B). This inclusion morphology was ubiquitous. It has been previously shown that IFN-γ, an inducer of a persistence-like phenotype in vitro, significantly constrains chlamydial growth in vitro [6]. For comparison (Figure 5C), acridine orange staining shows that inclusions in *Chlamydia*-infected cells treated with IFN-γ were stochastically reduced in size, and were also primarily filled with RBs, some of larger “aberrant” size, as has been reported.

Differences in ultrastructural observations of *Chlamydia* during variously induced persistent conditions have been noted previously [14]. A large inclusion containing both opaque RBs (1 µm) and smaller, darker EBs (0.3 µm) are observed by transmission electron microscopy in untreated cells infected with *C. trachomatis* (Figure 5D). However, inclusions in EHNA- and adenosine-treated cells contained sparsely-populated inclusions (Figure 5E).

## Discussion

Bacterial persistence has been studied in varied circumstances [15], [16], but may have unique characteristics for intracellular bacterial pathogens [14], [17]. Pathogens which are obligately intracellular typically have significantly reduced genome size and would be expected to have a lower capacity to withstand disruptions to their microenvironment, possibly developing alternate growth or subsistence mechanisms. Many alterations to the microenvironment during chlamydial infection lead to the initiation of a persistence program during which the chlamydiae experience severely diminished growth and modifications of both bacterial and inclusion development, although causative stimulants and the ultrastructural manifestations can vary [14].

One mode of sequestration from the adaptive immune response is the formation of granulomas in the host tissue, which may be initiated or altered by the pathogen [18]. Granulomas are typically observed during *C. trachomatis* infection, and become more abundant under conditions where inflammation is enhanced [19]. Notably, granulomas were absent following chlamydial infection of IL-10 knockout mice which had a more dominant TH1 type immune cell response than wild-type mice [20]. During sequestration within granulomas, the bacteria may not have sufficient nutrient accessibility for growth, being isolated from substantial blood flow under tissue conditions which have been shown to be hypoxic during infections with another intracellular pathogen, *Mycobacterium tuberculosis* [21], [22], which affects not only replicative capacity but also sensitivity to different modes of antimicrobial therapy.

Members of the AR family have been shown to play critical roles in the host response to microbial pathogens [7], [9], [23]. The A2a AR has been well studied in the context of being the dominant receptor in the behavioral response to caffeine, and for its role in regulation of immune function and inflammation where adenosine serves as a ‘danger signal’ [7]. The A2b AR has a lower affinity for adenosine relative to A2a which suggests that A2b should function in areas of increased adenosine concentration. Responses to adenosine in epithelial cells, including primary epithelial cells [24], have been previously characterized, with both A2a [25] and A2b [26] contributing to cell responses depending on tissue type.

While an increase in the intracellular cAMP concentration led to diminished growth of *C. trachomatis* in epithelial cells, this was not mediated by cAMP-dependent protein kinase (PKA). Other signaling pathways dependent on cAMP, but independent of PKA, have been characterized and can be activated following A2b stimulation, including cAMP-stimulated GDP exchange factor (EPAC1 and EPAC2) pathways [27]. The A2a adenosine receptor acts almost exclusively through cAMP modification, but the A2b receptor can also stimulate phospholipase C (PLC), and phosphoinositide 3-kinase (PI3K), which may contribute to the effects characterized here [28], [29]. Future work will further identify the downstream mediators of the cAMP-dependent, PKA-independent pathway responsible for chlamydial growth inhibition.

The A2b AR is upregulated during hypoxia, and has an HIF-1α binding site in its promoter region [9]. *C. pneumoniae* has been shown to directly disrupt function of HIF-1a during infection of epithelial cells in vitro [30], which indicates that this lung pathogen may attempt to counteract the effects of A2b expression in actively infected cells. However, this also suggests that hypoxia has been an important obstacle for *C. pneumoniae* survival, given that the minimalist genome of *Chlamydia* species maintains a commitment to specific disruption of this signaling pathway.

AR signaling, particularly through the A2b receptor, may play an important role in disease progression or abrogation during infections that result in local hypoxia, such as in granulomas caused by chlamydial infection [19]. Clarification of the role played by adenosine-mediated signaling may thus shed light on the mechanisms underlying persistence during infection with *Chlamydia* and other intracellular pathogens.

## Materials and Methods

### Cells, Bacteria, and Reagents

HeLa229 cells (American Type Culture Collection, Manassas, VA) were cultured in a humidified incubator at 37°C with 5% CO_2_ in Dulbecco's modified Eagle medium (DMEM) supplemented with 10% heat-inactivated fetal calf serum. The LGV/L2 strain of *C. trachomatis* was from Dr. Roger Rank (University of Arkansas, Little Rock, AR), and *C. trachomatis* serovar D was from Dr. David Nelson (Indiana University, Bloomington, IN). Adenosine (ADO), 5′-(N-ethylcarboxamido)adenosine (NECA), erythro-9-(2-Hydroxy-3-nonyl) adenine (EHNA), 8-bromoadenosine-3′, 5′-cyclic monophosphate (8-Br-cAMP), cycloheximide, and forskolin (FSK) were from Sigma (St. Louis, MO).

### Cell Culture and Infection

HeLa cells growing at 70% confluence in tissue culture plates (Costar) were infected with the LGV/L2 or D serovars (as indicated) of *C. trachomatis* at a multiplicity of infection (MOI) of 1.0, unless otherwise noted, and incubated at 37°C under 5% CO_2_ with treatments and media changes at the indicated times. The number of infectious chlamydial inclusion forming units was determined as previously described [31]. In brief, material from HeLa cells infected under different conditions and times was harvested from wells using a cell scraper, frozen at −80°C, thawed and thoroughly vortexed before titrating on 50% confluent HeLa cells for quantification by light microscopy or immunofluorescence on a widefield fluorescence microscope (Leica, Deerfield, IL, USA). Cells were stained with *C. trachomatis* genus antibodies from Argene (North Massapequa, NY), as described.

### cAMP Measurement

Intracellular cAMP levels were evaluated using a direct cAMP enzyme immunoassay (Assay Designs, Ann Arbor, MI), per the manufacturer's recommendations, including optional acytelation of samples.

### shRNA

MISSION® shRNA Lentiviral Transduction Particles for human A2a and A2b ADO receptors, as well as non-targeted shRNA (SHC002V), were acquired from SigmaAldrich, and used per manufacturer's recommendations for adherent cells. HeLa cells were transduced separately with each construct per the manufacturer's recommendations, and selected by addition of puromycin (Sigma) at 2 µg/ml. Cells expressing each construct were individually tested by quantitative PCR to evaluate knockdown of the target genes, and in our hands lentiviral transduction particles TRCN0000008043 for human A2a and TRCN0000065335 for A2b ADO receptors were most effective in HeLa cells. Sequences for these constructs are available from Sigma online.

### siRNA

Two sequences of siRNA targeting PKA C-α, and non-targeted siRNA, were obtained from Cell Signaling Technology (Danvers, MA), and transfected into HeLa cells using Lipofectamine 2000 (Invitrogen, Carlsbad, CA) per both manufacturers' recommendations. mRNA depletion (qPCR) and protein depletion (Western blot) were verified 30 hours post transfection, the time at which treatment was applied in functional assays.

### Western Blotting

Samples were lysed using RIPA Lysis Buffer (Millipore), protein quantities evaluated by the Bradford assay, and loaded onto SDS-PAGE 12% gel, and then transferred to a polyvinylidene difluoride membrane (Millipore). Blots were blocked for 1 h with 5% (w/v) nonfat dried milk in TBST. The membrane was incubated overnight at 4°C with with rabbit anti-PKA C-α (Cell Signaling Technology, Danvers, MA) and then incubated again with conjugated anti-rabbit IgG horseradish peroxidase (Millipore). Immunoreactive proteins were detected with ECL Plus Western Blotting Detection Reagents (Amersham) using a gel doc system (Biorad).

### Microscopy

For Acridine Orange staining, cells were fixed in acid alcohol (1 part glacial acetic acid, and 1 part absolute ethanol (Fisher Scientific)) for 10 minutes, and then rinsed 3 times with McIlvaines buffer (pH 3.8, 18.7 g Na_2_HPO_4_-7H_2_O,13.9 g citric acid-H_2_O, water to 200 ml). Coverslips with fixed cells were then stained with 0.01% Acridine Orange (Molecular Probes, Eugene, OR) for 4 minutes, washed 3 times with McIlvaines buffer. Slides were viewed on the widefield fluorescence microscope. For electron microscopy cell samples, HeLa cells growing at 70% confluence in tissue culture plates (Costar) were infected with the LGV/L2 strain of *C. trachomatis* at an MOI of 1.0 and incubated at 37°C under 5% CO_2_ with treatments and media changes at the indicated times. The cells were fixed in 2.5% glutaraldehyde (Ted Pella Inc., Redding, CA) for 1 hour, rinsed in PBS, then pelleted and embedded in warm agar. The tissue was then immersed in 4% OsO_4_ (Ted Pella) for 1 hour, followed by 3 rinses in PBS. Cell pellets were stained en bloc with 0.5% uranyl acetate overnight, then rinsed 3 times in distilled water. The pellets were dehydrated through a series of ethanol concentrations (25%, 50%, 75%, 95%, and 100%) for 10 minutes at each concentration, then embedded in resin (Ted Pella) at 1∶2 resin:ethanol overnight, 1∶1 resin:ethanol for 8 hours, 2∶1 resin:ethanol overnight, and 100% resin for 8 hours. The tissue was finally moved to new 100% resin in conical BEEM capsules (Ted Pella) and hardened at 65°C for 24 hours. Ultra thin sections (90 nm) were obtained using an ultramicrotome (Power Tome-XL, RML), stained with uranyl acetate and lead citrate in sequence, and observed with a FEI Tecnai 12 Transmission electron microscope.

### RNA Extraction

Total RNA was extracted using Trizol® Reagent (Invitrogen) following the manufacturer's instructions. The total RNA was quantified by measuring the optical density with a NanoDrop ND-1000 Spectrophotometer (NanoDrop, Wilmington, DE).

### cDNA Synthesis

Two µg of total RNA were reverse-transcribed at 42°C using TaqMan reverse trancriptase (Applied Biosystems) and Oligo(dT) according to the manufacturer's recommendations.

### Quantitative PCR with SYBR Green

For each transcript, a standard curve was constructed using the purified PCR product generated for each specific primer pair. Each PCR reaction utilized Brilliant® SYBR® Green Master Mix (Stratagene), and consisted of 25 µl containing 1 µl of cDNA and 5 pmol of each primer. A non-template negative control to check for primer-dimerization was run for each primer pair. The real-time qPCR was run on an MX3000p (Stratagene). The cycling conditions were 1 cycle of denaturation at 95°C/10 min, followed by 40 three-segment cycles of amplification (95°C/30 sec, 55°C/1 min, 72°C/30 sec), where the fluorescence was automatically measured during PCR and one three-segment cycle of product melting (95°C/1 min, 55°C/30 sec, 95°C/30 sec). The baseline adjustment method of the Mx3000 software was used to determine the C_t_ in each reaction. A melting curve was constructed for each primer pair to verify the presence of one gene-specific peak and the absence of primer dimerization. All samples were amplified in triplicates and the mean was used for further analysis. Primer sequences for the human ADO A2a receptor were: 5′-GGAGTTTGCCCCTTCCTAAG-3′, forward primer; and 5′- CTTCTCCCAACGTGACTGGT-3′, reverse primer. For the ADO A2b receptor, the primers were: 5′-GCTCCATCTTCAGCCTTCTG-3′, forward primer; and 5′-ACCCAGAGGACAGCAATGAC-3′, reverse primer. For *C. trachomatis* 16s rRNA the primers were: 5′-CCCGAGTCGGCATCTAATAC-3′ forward primer, and 5′-CTACGCATTTCACCGCTACA-3′ reverse primer. Human GAPDH was used as the housekeeping gene control for AR expression analysis, for which the primers were: 5′-CGACCACTTTGTCAAGCTCA-3′ forward primer, and 5′-AGGGGAGATTCAGTGTGGTG-3′.

### Analysis of mRNA Expression for Adenosine Receptors

In order to determine whether HeLa cells express the adenosine receptor genes (A1, A2a, A2b, A3), PCR amplification was carried out with primers specific for the human genes. The sequences of the primers for GAPDH, A2a, and A2b were described in the qPCR methods, and the A1 and A3 sequences used were as follows A1, forward primer, 5′-TCAGTCCAGTCCTCACATGC -3′; reverse primer, 5′-GTGGAGGGACCACACTCTGT-3′ (amplicon expected size, 200 bp); A3, forward primer, 5′-TACTGGTGCCGAGGCTATTT-3′; reverse primer, 5′-TGGTCAGGCAGGACATAGTG-3′. The PCR cycling protocol for all primers was 94°C for 30 seconds, 50°C for 30 seconds and 72°C for 30 seconds. The protocol was repeated for 40 cycles and included an initial 5 min enzyme activation step at 94°C and a final 10 min extension step at 72°C. PCR products were separated by electrophoresis on a 2% agarose gel and visualized by ethidium bromide staining.

## Supporting Information

Figure S1 Adenosine is released from hypoxic cells, and adenosine levels are also elevated by processing of released ATP from stressed cells (via CD73 and CD39 ecto-enzymes). A2b receptor expression is also increased in tissues with low oxygen, mediated by the hypoxia-inducible factor-1alpha (HIF-1alpha) transcription factor. Ligation of the A2b adenosine receptor during infection with the intracellular pathogen C. trachomatis causes reversible inhibition of bacterial development. Exposure to elevated extracellular adenosine may lead to persistent infections in vivo.
(0.10 MB TIF)

Figure S2 Treatment with NECA, an AR agonist, reversibly inhibits growth of Chlamydia. HeLa cells were infected with *C. trachomatis* serovar L2 at an MOI of 1, followed by treatment with an equivalent volume of vehicle alone (Control) or with 100 uM NECA at 20 hours post infection (hpi). Samples were harvested at the indicated times for quantification of reinfectious yield (IFU/ml) on new HeLa cell monolayers. Treatment with NECA caused a 90% or greater reduction in reinfectious yield at times shortly after treatment (25 and 30 hpi), but reinfectious yield was similar in Control and NECA-treated samples at subsequent timepoints. The values show averages and S.D. from 3 samples of a representative experiment, and represent results obtained from two independent experiments.
(0.10 MB TIF)

Figure S3 cAMP levels in HeLa cells were evaluated using an enzyme immunoassay. Cells were treated with 50 uM adenosine (Ado) in the presence or absence of EHNA (25 uM), as indicated, over the displayed time-course. Samples were lysed in 0.1 M HCl with 0.1% Triton X-100, following the manufacturer's protocol, which included sample acetylation. The values show averages and S.D. from 3 independent experiments. (n = 3, *, P<0.05, for EHNA plus adenosine treated cells compared to cells treated with adenosine alone.)
(0.06 MB TIF)

Figure S4 Expression of adenosine receptor mRNA in HeLa cells. PCR was performed as indicated in Materials and Methods. Expected amplicon sizes were: GAPDH, 203 bp; A1, 121 bp; A2a, 72 bp; A2b, 121 bp; A3, 124 bp. The expression levels are representative of two experiments prepared from separate cell samples on separate dates.
(0.05 MB TIF)

Figure S5 HeLa cells were infected with *C. trachomatis* serovar D at an MOI of 1, followed by treatment with an equivalent volume of vehicle alone (Control) or 25 uM EHNA and 50 uM adenosine 1 hpi. Samples were harvested at 42 hpi for quantification of reinfectious yield (IFU/ml) on new HeLa cell monolayers. Prolonged exposure to adenosine caused an 80% or greater reduction in reinfectious yield. The values show averages and S.D. from triplicates of a representative experiment, and represent results obtained from two independent experiments.
(0.06 MB TIF)

Figure S6 Depletion of PKA C-alpha in HeLa cells. siRNA targeting PKA C-alpha was transfected into HeLa cells, and mRNA levels of PKA C-alpha 30 hours post transfection were compared to nontarget siRNA controls and untreated cells. The values show averages and S.D. from three independent experiments.
(0.06 MB TIF)

Figure S7 Effect of extracellular adenosine (Ado) does not require new host-cell protein synthesis. HeLa cells were infected with *C. trachomatis* serovar L2 at an MOI of 1, followed by treatment as indicated at 1 hpi. Concentrations of reagents were: cycloheximide, 1 ug/mL; EHNA, 25 uM; adenosine, 50 uM. Total RNA was isolated at 24 hpi for quantification of chlamydial 16s rRNA production using qPCR as indicated in Materials and Methods. The values shown are relative to control values and are representative of two independent experiments.
(0.07 MB TIF)

Figure S8 Reversible effect of adenosine (Ado) on chlamydial infection. HeLa cells were infected with *C. trachomatis* serovar L2 at an MOI of 1, followed by treatment with an equivalent volume of vehicle alone (control) or 25 uM EHNA and 50 uM adenosine 1 hpi (treated and recovery). Media was refreshed in all samples at 24 hpi, and EHNA and adenosine added again to the “treated” samples only. Samples were harvested at 44 hpi and 72 hpi for quantification of reinfectious yield (IFU/ml) on new HeLa cell monolayers. Reinfectious yield recovered to near control values within 48 hours of discontinuation of elevated adenosine exposure. The values show averages and S.D. from triplicates of a representative experiment, and represent results obtained from two independent experiments.
(0.05 MB TIF)

## References

[1] Belland R, Ojcius DM, Byrne GI (2004). *Chlamydia*.. Nature Rev Microbiol.

[2] Moulder JW (1991). Interaction of chlamydiae and host cells in vitro.. Microbiol Rev.

[3] Wyrick PB (2000). Intracellular survival by *Chlamydia*.. Cell Microbiol.

[4] Bavoil PM, Hsia R-c, Ojcius DM (2000). Closing in on *Chlamydia* and its intracellular bag of tricks.. Microbiol.

[5] Roan NR, Starnbach MN (2008). Immune-mediated control of Chlamydia infection.. Cell Microbiol.

[6] Beatty WL, Morrison RP, Byrne GI (1994). Persistent chlamydiae: from cell culture to a paradigm for chlamydial pathogenesis.. Microbiol Rev.

[7] Sitkovsky MV, Ohta A (2005). The ‘danger’ sensors that STOP the immune response: the A2 adenosine receptors?. Trends Immunol.

[8] Eltzschig HK, Ibla JC, Furuta GT, Leonard MO, Jacobson KA (2003). Coordinated adenine nucleotide phosphohydrolysis and nucleoside signaling in posthypoxic endothelium: role of ectonucleotidases and adenosine A2B receptors.. J Exp Med.

[9] Kong T, Westerman KA, Faigle M, Eltzschig HK, Colgan SP (2006). HIF-dependent induction of adenosine A2B receptor in hypoxia.. Faseb J.

[10] Sitkovsky MV, Lukashev D, Apasov S, Kojima H, Koshiba M (2004). Physiological control of immune response and inflammatory tissue damage by hypoxia-inducible factors and adenosine A_2A_ receptors.. Annu Rev Immunol.

[11] Ward ME, Salari H (1982). Control mechanisms governing the infectivity of Chlamydia trachomatis for hela cells: modulation by cyclic nucleotides, prostaglandins and calcium.. J Gen Microbiol.

[12] Darzynkiewicz Z (1990). Differential staining of DNA and RNA in intact cells and isolated cell nuclei with acridine orange.. Methods Cell Biol.

[13] Hoare A, Timms P, Bavoil PM, Wilson DP (2008). Spatial constraints within the chlamydial host cell inclusion predict interrupted development and persistence.. BMC Microbiology.

[14] Hogan RJ, Mathews SA, Mukhopadhyay S, Summersgill JT, Timms P (2004). Chlamydial persistence: beyond the biphasic paradigm.. Infect Immun.

[15] Balaban NQ, Merrin J, Chait R, Kowalik L, Leibler S (2004). Bacterial persistence as a phenotypic switch.. Science.

[16] Kussell E, Kishony R, Balaban NQ, Leibler S (2005). Bacterial persistence: a model of survival in changing environments.. Genetics.

[17] Young D, Hussell T, Dougan G (2002). Chronic bacterial infections: living with unwanted guests.. Nat Immunol.

[18] Russell DG (2007). Who puts the tubercle in tuberculosis?. Nat Rev Microbiol.

[19] Perfettini JL, Ojcius DM, Andrews CW, Korsmeyer SJ, Rank RG (2003). Role of proapoptotic BAX in propagation of Chlamydia muridarum (the mouse pneumonitis strain of Chlamydia trachomatis) and the host inflammatory response.. J Biol Chem.

[20] Yang X, Gartner J, Zhu L, Wang S, Brunham RC (1999). IL-10 gene knockout mice show enhanced Th1-like protective immunity and absent granuloma formation following Chlamydia trachomatis lung infection.. J Immunol.

[21] Via LE, Lin PL, Ray SM, Carrillo J, Allen SS (2008). Tuberculous granulomas are hypoxic in guinea pigs, rabbits, and nonhuman primates.. Infect Immun.

[22] Tsai MC, Chakravarty S, Zhu G, Xu J, Tanaka K (2006). Characterization of the tuberculous granuloma in murine and human lungs: cellular composition and relative tissue oxygen tension.. Cell Microbiol.

[23] Deaglio S, Dwyer KM, Gao W, Friedman D, Usheva A (2007). Adenosine generation catalyzed by CD39 and CD73 expressed on regulatory T cells mediates immune suppression.. J Exp Med.

[24] Lazarowski ER, Tarran R, Grubb BR, van Heusden CA, Okada S (2004). Nucleotide release provides a mechanism for airway surface liquid homeostasis.. J Biol Chem.

[25] Sun Y, Wu F, Sun F, Huang P (2008). Adenosine promotes IL-6 release in airway epithelia.. J Immunol.

[26] Huang P, Lazarowski ER, Tarran R, Milgram SL, Boucher RC (2001). Compartmentalized autocrine signaling to cystic fibrosis transmembrane conductance regulator at the apical membrane of airway epithelial cells.. Proc Natl Acad Sci U S A.

[27] Fang Y, Olah ME (2007). Cyclic AMP-dependent, protein kinase A-independent activation of extracellular signal-regulated kinase 1/2 following adenosine receptor stimulation in human umbilical vein endothelial cells: role of exchange protein activated by cAMP 1 (Epac1).. J Pharmacol Exp Ther.

[28] Schulte G, Fredholm BB (2003). Signalling from adenosine receptors to mitogen-activated protein kinases.. Cell Signal.

[29] Wang D, Sun Y, Zhang W, Huang P (2008). Apical adenosine regulates basolateral Ca2+-activated potassium channels in human airway Calu-3 epithelial cells.. Am J Physiol Cell Physiol.

[30] Rupp J, Gieffers J, Klinger M, van Zandbergen G, Wrase R (2007). Chlamydia pneumoniae directly interferes with HIF-1alpha stabilization in human host cells.. Cell Microbiol.

[31] Darville T, Welter-Stahl L, Cruz C, Abdul Sater AA, Andrews CW (2007). Effect of the purinergic receptor P2X_7_ on *Chlamydia* infection in cervical epithelial cells and vaginally-infected mice.. J Immunol.

